# Supplementary figures and images for: Blood and brain gene expression signatures of chronic intermittent ethanol consumption in mice
Source: PLoS Comput Biol. 2022 Feb 17;18(2):e1009800. doi: 10.1371/journal.pcbi.1009800 (PMC8853518; doi:10.1371/journal.pcbi.1009800)

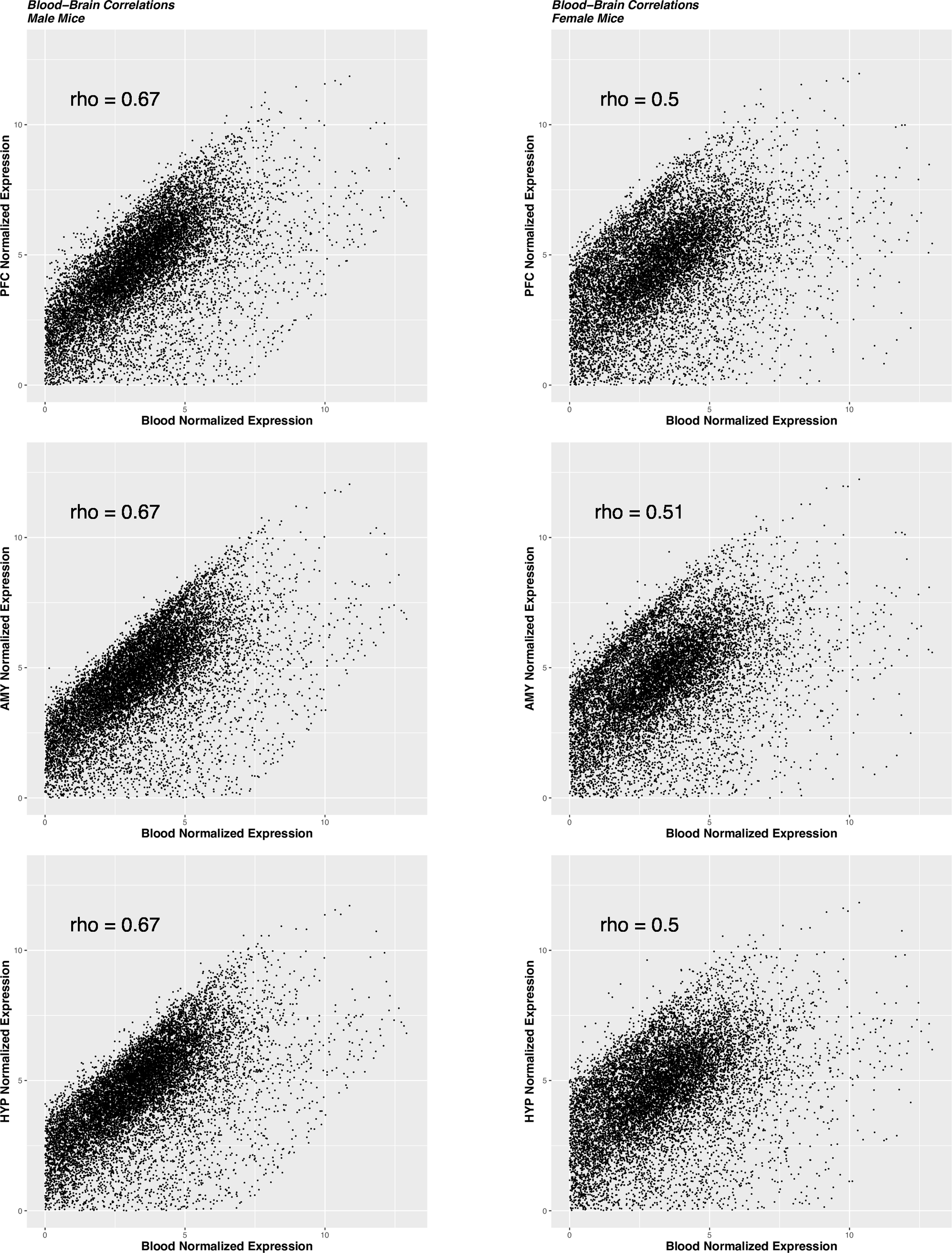

Supplement: S1 Fig — The scatterplots display the relationship between blood (x-axis) and brain (y-axis) mean gene expression levels for male (left) and female (right) mice. Each point in the scatterplot represented a gene, and the normalized expression level of the gene averaged across subjects (irrespective of treatment) are plotted. We then calculated the Spearman correlation coefficient between blood and brain normalized gene expression levels, and this value is displayed in the plots (rho). (TIF) [file pcbi.1009800.s001.tif]

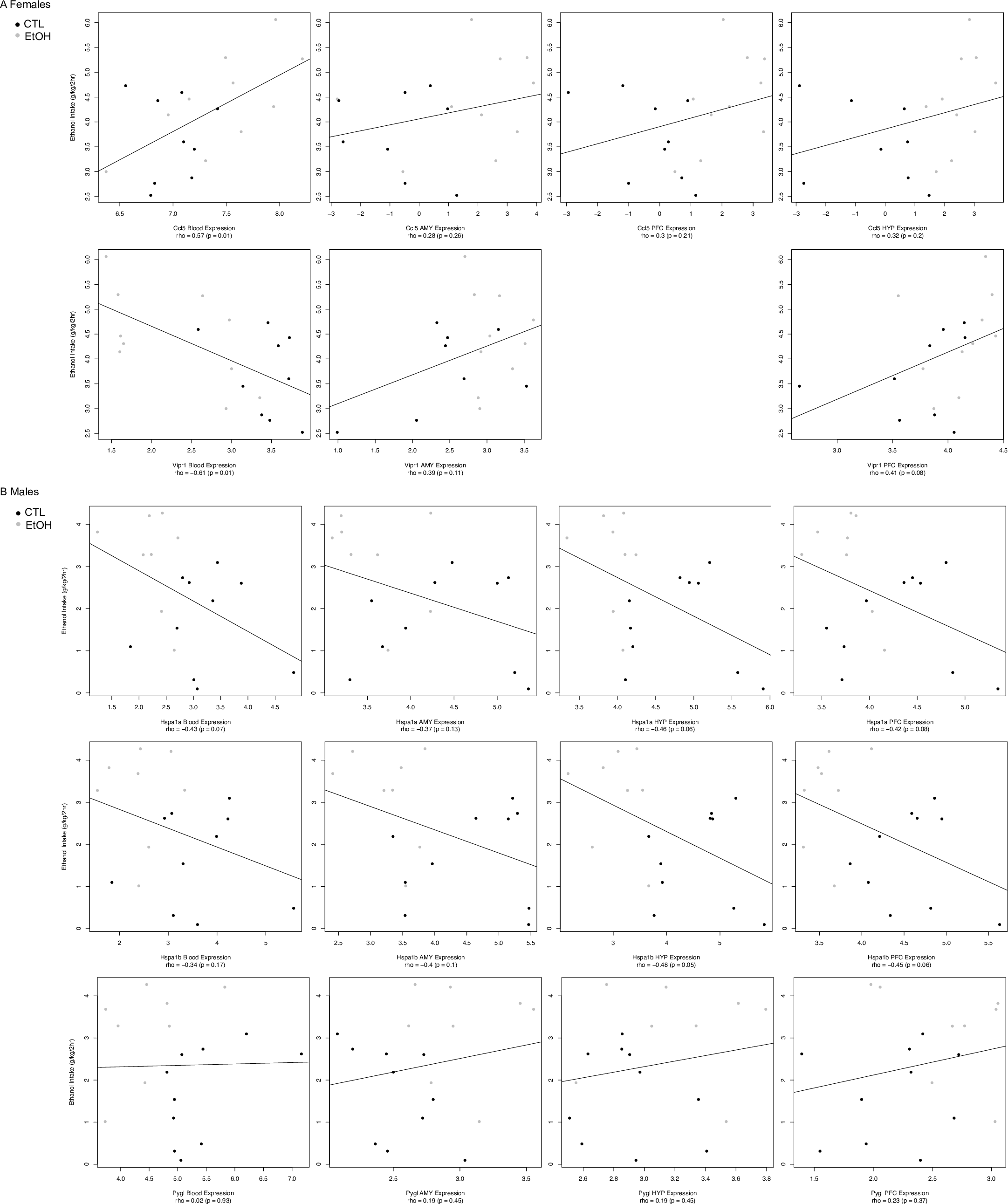

Supplement: S2 Fig — The scatterplots display the relationship between alcohol consumption in the final limited access two bottle choice drinking test (y-axis) and blood or brain gene expression levels (x-axis) for the genes presented in Fig 4 for female (A) and male (B) mice. Each point in the scatterplot represented a subject. We then calculated the Pearson correlation coefficient between the normalized gene expression levels and alcohol intake and this value and associated p-value is displayed under the x-axis. (TIF) [file pcbi.1009800.s002.tif]
