# Supplementary material for: Blood and brain gene expression signatures of chronic intermittent ethanol consumption in mice
Source: PLoS Comput Biol. 2022 Feb 17;18(2):e1009800. doi: 10.1371/journal.pcbi.1009800 (PMC8853518; doi:10.1371/journal.pcbi.1009800)
Supplement: Attachment — Submitted filename: Responses_to_Reviewers_final.docx [file pcbi.1009800.s012.docx]

November 22, 2021

RE: Manuscript #PCOMPBIOL-D-21-01250

Dear Dr. Granlund and Dr. Ioshikhes,

Thank you very much for reviewing our manuscript. We are pleased to resubmit the revised version of Ms. Ref. No.: PCOMPBIOL-D-21-01250 "Blood and brain gene expression signatures of chronic intermittent ethanol consumption in mice" for publication in PLOS Computational Biology. The tracked changes in the revised manuscript represent sentences or sections that were revised in response to the Reviewer’s comments. We appreciate the constructive criticisms of the Editors and Reviewers and have addressed each of their concerns as outlined below.

We thank you and the Reviewers for your time and careful consideration. We believe that the manuscript has been greatly improved by addressing these concerns and comments. We hope that you find our responses satisfactory and that the manuscript is now acceptable for publication.

Sincerely,

Laura Ferguson, Ph.D.

**Reviewer #1: This manuscript by Ferguson and colleagues performs a rigorous comparison of total and ethanol-responsive gene expression profiles in blood versus several brain regions in C57BL/6J mice that have been treated with a chronic intermittent ethanol vapor + consumption model of ethanol exposure. While a number of other studies have done somewhat similar work for other disease states or with postmortem tissue, this is apparently the first well-controlled animal model study to conduct such whole genome profiling of brain vs. blood with ethanol treatment. The experimental design and methodology are well described and the results are presented in a thorough and clear fashion. The study presents a comprehensive analysis at the level of individual genes, expression networks, biologic functional groups, and a “network of networks” approach to assess overlap of brain vs. blood expression networks. Finally, the authors also present the use of 3 different predictive models to assess the ability of blood expression patterns to predict the treatment status of the mice (alcohol exposed vs. control). Overall, this is a very thorough and well-conducted study of importance to the field in terms of identifying approaches for blood biomarker utilization in assessment of alcohol use disorder clinical status. The approaches utilized should lead to future important work with potential clinical implications.

Weaknesses of the work were few and included:**

We thank the Reviewer for taking the time to carefully evaluate the manuscript and for appreciating the value and importance of the work.

**1) The classification analysis appeared impressive but, in retrospect, seems somewhat superficial with limited direct clinical application as currently developed, although future iterations may be very fruitful. Predicting whether a mouse has been exposed to weeks of high dose ethanol vapor, based upon blood expression patterns, is a rather low bar and the clinical equivalent seems even less significant. It is usually very clinically evident, by history, physical exam or routine laboratory studies, that an individual is a severe case of alcohol use disorder. Whether, the blood expression profiles might detect milder forms of alcohol use disorder and thus be more clinically useful would require either an additional control group (e.g. no ethanol intake at all) or a larger number of animals to allow analysis of low vs. high drinkers. The investigators should at least include some of these issues in the Discussion.**

We agree with the Reviewer and have added the following text to the Discussion section to address this topic.

“The mice in this study have been exposed to weeks of high dose ethanol vapor and would likely represent severe cases of AUD. These cases are usually clinically evident by patient history and physical exam and would not necessitate a molecular diagnostic tool. However, social stigma surrounding alcoholism (like most psychiatric disorders) remains a barrier to treatment. An objective molecular diagnostic tool (e.g., a “blood test”) would potentially mitigate stigma and encourage patients to seek diagnosis and treatment for this disorder. Additionally, blood expression profiles might detect milder forms of AUD or those at high-risk for developing AUD. The former could be investigated in future studies that include an additional ethanol-naïve control group or a larger number of animals to allow analysis of low versus high drinkers. The latter could be addressed in future studies by profiling the blood of ethanol-naïve genetic rodent models of AUD-risk traits (e.g., high alcohol preference or consumption, propensity for binge-like drinking), or by analyzing the blood transcriptome before and after CIE treatment and correlating gene expression changes with the amount of drinking escalation within subjects. The blood transcriptome varies between people, but it is relatively stable for an individual across time (1) which is encouraging and suggests that repeated blood sampling could be used for personalized medicine approaches. Studies that analyze gene expression profiles in blood sampled at multiple time points throughout the addiction cycle (including before alcohol exposure) will be critical in exploring the clinical utility of blood gene expression profiles for AUD.”

**2) It is surprising that despite the extremely detailed analytical scheme and bioinformatics analysis, that the investigators did not include a co-analysis of their brain and blood network patterns vs. networks/genes that have been published for autopsied human tissue or GWAS studies on AUD or alcohol consumption. Such cross-species studies would add clinical relevance.**

We considered performing a co-analysis with human datasets; however, as the Reviewer noted, the goal was to perform a well-controlled animal model study to conduct whole genome profiling of brain vs. blood with ethanol treatment, and we respectfully felt that such an analysis was beyond the scope of the current study design. The current study is extensive and contains 8 large and novel datasets (4 tissues for both sexes), which advances the understanding of the molecular actions of CIE-induced alcohol dependence. Additionally, we recently published a Review of 17 postmortem human transcriptome datasets (2), and do not think adding a comparison analysis in the present study would further contribute to the existing literature. This work adds to the canon of knowledge as it provides the first analysis of expression changes in blood following CIE, and the within-subjects design enabled us to establish a link between blood and brain responses to CIE.

We are currently collecting human blood samples from patients with AUD and controls to determine whether blood gene expression profiles from these subjects can be used to classify individuals as alcohol dependent or non-dependent. While brain tissue will not be collected, we are collecting blood samples at additional time points (including baseline levels). We are hopeful that this study (which is in the data collection phase) will provide more clinically relevant information.

**3) Some of the individual gene correlations in blood vs. brain were quite impressive (Fig. 4) and a number of these were regulated by ethanol vapor exposure. The multiple heat shock proteins are particularly striking as they have been identified as ethanol-responsive genes dating back to the early 1990’s. It would be an additional piece of useful information to see a plot of the expression for some of these genes (in brain or blood) vs. the ethanol consumption levels for ethanol control or vapor-exposed animals – to identify the impact of oral consumption vs. just the vapor.**

Thank you for this suggestion. We performed this analysis and included the following text in the Results section and provided the scatter plots as a Supplemental Figure (Figure S2). Additionally, we now provide an interactive RShiny app where users may search for a gene of interest and see its correlation with alcohol consumption (https://lauraferguson.shinyapps.io/blood_brain/).

“Some of the correlated genes were also differentially expressed between CIE and Air mice in both blood and brain (Tables 2 - 4). For males, *Hsp1a* and *Hsp1b* were correlated between blood and each brain area and differentially expressed in blood and all brain regions, as was *Pygl* (except for hypothalamus) (Figure 4). Blood and brain levels of *Hspa1a* and *Hspa1b* tended to be negatively correlated with alcohol consumption in the last 2BC drinking test, while *Pygl* had weak correlations with alcohol intake (Figure S2). For females, *Ccl5* was correlated between blood and each brain area and differentially expressed in blood and all brain regions, as was *Vipr1* (except for hypothalamus) (Figure 4). Blood expression levels of *Ccl5* were positively correlated with alcohol intake, while blood expression levels of *Vipr1* were negatively correlated with alcohol intake (Figure S2). We created an R Shiny app where interested readers can see the correlation between voluntary ethanol intake levels and the expression level of any gene of interest (https://lauraferguson.shinyapps.io/blood_brain/).”

**4) Although perhaps a minor semantic issue, the extensive use of the term “alcohol dependent” in describing the animals exposed to ethanol vapor (CIE) is somewhat unsettling. The terminology “alcohol dependence” usually refers to a clinical state consisting not just of the presence of withdrawal symptoms upon ethanol abstinence, but of obsessive ethanol seeking behavior, amongst other clinical signs. The CIE mice do show some increased ethanol consumption and other investigators have shown occurrence of increased withdrawal seizures and evidence of tolerance – but this reviewer is not certain that the term “alcohol dependent” is entirely appropriate.**

We thank the reviewer for highlighting this issue. Chronic Intermittent Ethanol exposure and withdrawal (CIE) is a well-validated and commonly used mouse model to produce alcohol dependence which is why we used the term “alcohol dependent” in the manuscript. However, we understand the Reviewer’s concern regarding the use of the term “alcohol dependent” and are grateful for the comment as we never wish to overstate the clinical implications of our findings. We have therefore replaced the term “alcohol dependent” with “CIE”, and “non-dependent” with “Air” throughout the manuscript.

**Reviewer #2: The submission entitled “Blood and brain gene expression signatures of chronic intermittent ethanol consumption” describes gene expression changes in alcohol-dependent and control mice in three brain regions, namely: prefrontal cortex, hypothalamus and amygdala as well as in blood; and analyses how such changes correlate between brain and blood using a within-subject approach. Their results start to address a gap in our knowledge about how blood gene expression profiles can be used to diagnose AUDs, and have the potential to aid in the development of personalized and more effective therapeutic interventions. The manuscript is well written, the limitations clearly stated, and the data was thoroughly analyzed; RNA sequencing information was made publicly available in Gene Expression Omnibus.
The following revisions are recommended:**

We thank the Reviewer for their encouraging words regarding thoroughness of the analysis, the writing, and the importance of the work. We tried to address each of the Reviewer’s concerns and hope it is satisfactory to the Reviewer.

**- Figure 2A is missing labels over: male AMY increase, female PFC decrease, male PFC increase, male HYP increase, female blood increase, male blood increase.**

There are no labels because there were no cell types significantly enriched in those datasets as noted in the Results section “In males, there were no cell type specific genes enriched in any of the up-regulated gene sets (Figure 2A).” We changed the wording in the Figure legend for clarification:

“If there were any significantly enriched cell type specific genes in the datasets (hypergeometric test; Bonferroni-corrected p < 0.05), these are indicated adjacent to the respective dataset. No label indicates that there were no cell type specific genes enriched in the dataset.”

**- In the introduction, explain the specific reasons why amygdala, hypothalamus and prefrontal cortex where chosen for this study. In the methods, please clarify what specific sub-regions where collected for each brain region, was the 1mm punch used to collect from all sub-regions or was there a focus on specific ones?**

We added the following to the Introduction:

“To fill this knowledge gap, we compared gene expression levels in whole blood to those in the amygdala, prefrontal cortex, and hypothalamus from CIE mice undergoing withdrawal. We chose these brain areas because of their importance in reward signaling and alcohol dependence (3, 4) and because gene expression responses in these brain areas have been shown to parallel those in blood under other conditions (5-10).”

We used the 1 mm punch to collect from all the sub-regions. We added this information to the Methods section.

“We performed bilateral micropunch sampling on a frozen stage (−20 to −25° C) using a 1.00 mm diameter punch (Stoelting Co., item #57397, Wood Dale, IL, USA) to include all sub-regions of the brain areas according to the stereotaxic atlas of Paxinos and Franklin (11).”

**- In the results section, Page 10, lines 14-18 (Figure 2A), please clarify neuronal changes in the female hypothalamus; what are the differences between the neuronal genes going up and those going down?.**

Both the up-regulated and down-regulated genes in the female hypothalamus were enriched with genes preferentially expressed in neurons. The specific neuronal genes that are up-regulated and down-regulated are shown in the Table below, and we now provide these results (and those for the other tissue areas) in the manuscript as a Supplementary Table (Table S2). There are synaptic markers (*Slc17a6, Slc17a8, Stmn2, Stx1a, Cplx2, Slc32a1*), neuropeptides (*Sst, Penk, Cck*), and glutamatergic signaling markers (*Slc17a6, Slc17a8, Gria1, Grm8*) that are both increased and decreased, and a GABA marker that is decreased (*Gad2*) in the hypothalamus. The list of neuron-specific genes used for the analysis was from the literature (12), and it includes many different neuronal subtypes. Therefore, the current analysis provides a broad, high-level view of cell types potentially implicated in the gene expression responses to CIE. Further studies would be required to determine the specific neuron sub-types involved.

| Female Hyp | Cell Type | CorrectedPvalues | NumGenes | Genes |
| --- | --- | --- | --- | --- |
| Down | neu | 7.80E-09 | 52 | Ankrd55, Ar, Atg9b, Caln1, Cckbr, Col19a1, Cplx2, Dgkg, Dlx1, Dlx2, Ebf2, Ecel1, Elfn1, Eml5, Erc2, Frmpd3, Gad2, Galntl6, Gm14204, Gm16485, Gm26871, Gpr26, Gria1, Grip1, Grm8, Hcn1, Iqsec3, Irs4, Kcnip1, Klhl1, Mrap2, Ndst4, Nrxn3, Peli3, Penk, Pgr, Rab27b, Rxfp3, Slc32a1, Slc35f4, Sst, Thsd7b, Tnfaip8l3, Trim46, Trpc4, Tspyl5, Unc5a, Unc5d, Usp29, Vstm2a, Wscd2, Zfp804b |
| Up | mic | 2.18E-08 | 46 | Adap2, Apobec3, Arhgap9, Cd52, Cd74, Cd86, Creg1, Cryl1, Ctc1, Ctss, Dok2, Edem1, Fcer1g, Fyb, H2-Aa, H2-DMa, H2-Eb1, Haao, Hexb, Hs6st1, Irak4, Irf5, Itgb2, Lag3, Lrrc3, Lst1, Ltc4s, Ly86, Mafb, Med12l, Pik3cd, Ppp1r18, Ptafr, Ptpn18, Ptprc, Rac2, Rcsd1, Rtn4rl1, Sla, Slc11a1, Slc36a1, Slc7a8, Spi1, Tmem119, Tyrobp, Vrk2 |
| Up | neu | 0.00159337 | 43 | AI593442, Ano5, Arhgap15, Atp6ap1l, Btbd11, Cacng3, Cck, Cdkl5, Cit, Clec2l, Ctxn2, Cx3cl1, Diras2, Elavl2, Epha8, Fam49a, Fxyd7, Galnt9, Gpr12, Hecw1, Htr1f, Kcnj9, L1cam, Lancl2, Mal2, Mlip, Necab1, Nell1, Nrip3, Osbpl3, Pcsk2, Pdp1, Pgbd5, Prkaa2, Rab3c, Rab6b, Rgs4, Rph3a, Slc17a6, Slc17a8, Stmn2, Stx1a, Sv2b |
| Up | end | 0.00408498 | 35 | Art3, Atp10a, AW112010, Bok, Casq2, Cnn2, Col4a3bp, Ctnnd1, Dap, Ddx60, Ecm1, Foxp1, Galnt15, Gata2, Gjc1, Gpr4, Ifi35, Iigp1, Lef1, Mapk12, Mrvi1, Paqr5, Plcb4, Prkch, Ptgfrn, Rasd1, Slc16a4, Slc16a9, Slc25a24, Slc7a5, Slco2a1, Tnfaip8l1, Uaca, Zap70, Zfp326 |
| Down | oli | 0.01851998 | 33 | Ablim2, Agap1, Ankib1, Arhgef10, B3galt5, Ccdc13, Cd82, Cdc37l1, Cercam, Cryab, D7Ertd443e, Dock10, Dock5, Gm23935, Gpr17, Lcorl, Nkx6-2, Pdlim2, Plcl1, Ppfibp2, Ppp1r14a, Ppp1r16b, Prkcq, Ralgds, Rcor2, Rhog, Rnf122, Sh3gl3, Slain1, Taf9b, Tmem98, Tulp4, Vmp1 |

**- In the introduction and methods, please clarify if the animals where drinking after the 4th CIE cycle and explain the reason tissue was collected 1 week after CIE, presumably to avoid having alcohol on-board at collection time?. Typically mice will still show increased ethanol consumption on the last test-drinking day, but if they have not been given access to alcohol for a whole week their withdrawal/motivation state can’t be extrapolated to (or assumed to be) that of a test-drinking week.**

The mice underwent a final ethanol/air vapor exposure and were euthanized one week following this exposure. We added the following to the Methods section for clarification:

“All mice then received one final 4-day vapor or air exposure (without 2BC testing). One week later, mice were euthanized and tissue collected for RNA sequencing.”

We chose the one-week time point for a few different reasons in addition to avoiding having alcohol on-board at collection time as the Reviewer noted. This is the time point when increased voluntary alcohol intake is the highest (13). Also, we wanted to target longer-term expression changes and avoid the volatile gene expression changes that occur in the few days immediately following vapor exposure (14). We added this reasoning to the Introduction:

“We assessed gene expression profiles after 1 week of alcohol withdrawal because this is when increased voluntary alcohol intake is highest (13). Additionally, the assessment of longer-term gene expression effects of ethanol avoided the confounds of acute ethanol intoxication and the volatile gene expression changes observed in early withdrawal (14, 15).”

**Discussion

- Although the main focus of this study is to identify gene expression signatures common between blood and brain, it would be important for the authors to discuss how the brain DEGs found in dependent mice compare to those in human alcohol-dependent individuals in those same regions, according to the literature.**

Please see the response to Reviewer #1’s second point above.

**- The discussion would be strengthened if the expectations for developing better, more personalized, pharmacological interventions using blood gene sequencing are described and how the current results contribute to that end. How could this technology be used to identify individuals with high risk of AUDs, and help develop prophylactic interventions for the development of dependence?. Future studies, could analyze blood RNA seq before and after CIE treatment and correlate gene expression changes with the amount of drinking escalation within subjects.**

We agree and have substantially modified the Discussion section in response to this and Reviewer #1’s comments:

“Routine blood testing has long been a part of medical care. Genomic profiles in blood could be used to provide data-driven diagnosis of AUD, stratify the heterogeneous AUD patient population for clinical trials, select optimal therapy, determine whether a patient has AUD risk, and monitor the efficacy of the patient’s medications. Here we performed a well-controlled animal model study to conduct whole genome profiling of brain and blood with ethanol treatment. An important question this study addressed was whether blood gene expression signatures could predict CIE and Air mice. The highly predictive performance of the classifiers suggests that there is valuable diagnostic information in blood gene expression for CIE-induced alcohol dependence, even one week since the last alcohol exposure. Different features were selected by the different classification methods, which is consistent with the literature (16) and expected because the methods employ different techniques for the classification task and the features in gene expression datasets are highly correlated. These results confirm that good performance in the classification task can be achieved using different sets of features. That (and the dynamic nature of gene expression) might make it challenging to select a consistent panel of biomarkers and it will likely be necessary to incorporate other sources of information into a diagnostics screen. There were however several features ranked as highly important for achieving good performance in multiple models. This included *Gzma*, *Apol11a*, *Gatad2a*, *Hsd11b1*, and *Ankle1* for females and *Plet1*, *Hspa5*, *Cebpb,* and *Cxcr2* for males. *Cebpb* and *Cxcr2* are especially interesting considering the importance of immune molecules in responses to alcohol (discussed below). However, immune-responsive molecules may not be ideal biomarkers given that immune responses can be initiated by infections or other environmental perturbations.

AUROC measures the quality of the model's predictions irrespective of what classification threshold is chosen. The classification threshold strikes a balance between false positive and false negative rates, and the optimal choice would likely depend on the setting and purpose (e.g. primary care screening for problematic drinking versus criminal justice settings) (17). Moreover, the predictive value of these classifiers will depend on the prevalence of the disorder in the population tested. A population with low prevalence of the disorder (for example, in primary care screening for AUD) results in an increase in false positive tests, whereas a higher prevalence rate (e.g., in an addiction clinic) yields more false‐negative tests (17). Here in the controlled experimental conditions the prevalence was 50/50. Thus, our findings represent important first steps in identifying novel biomarkers for AUD, but more research (including human validation) will be required before genes selected as discriminatory features could become viable biomarkers for AUD.

The mice in this study have been exposed to weeks of high dose ethanol vapor and would likely represent severe cases of AUD. These cases are usually clinically evident by patient history and physical exam and would not necessitate a molecular diagnostic tool. However, social stigma surrounding alcoholism (like most psychiatric disorders) remains a barrier to treatment. An objective molecular diagnostic tool (e.g., a “blood test”) would potentially mitigate stigma and encourage patients to seek diagnosis and treatment for this disorder. Additionally, blood expression profiles might detect milder forms of AUD or those at high-risk for developing AUD. The former could be investigated in future studies that include an additional ethanol-naïve control group or a larger number of animals to allow analysis of low versus high drinkers. The latter could be addressed in future studies by profiling the blood of ethanol-naïve genetic rodent models of AUD-risk traits (e.g., high alcohol preference or consumption, propensity for binge-like drinking), or by analyzing the blood transcriptome before and after CIE treatment and correlating gene expression changes with the amount of drinking escalation within subjects. The blood transcriptome varies between people, but it is relatively stable for an individual across time (1) which is encouraging that repeated blood sampling can be used for personalized medicine approaches. Studies that analyze gene expression profiles in blood sampled at multiple time points throughout the addiction cycle (including before alcohol exposure) will be critical in exploring the clinical utility of blood gene expression profiles for AUD.”

**Reviewer #3: In this manuscript, the authors profiled and analyzed transcriptomes of three brain regions and blood in C57BL/6J mice one week after chronic intermittent ethanol (CIE) exposure. They found the transcript level between blood and three brain regions is highly preserved. Despite the small overlap between blood and brain DEGs, considerable overlap at gene network level was observed. The authors then used three machine-learning algorithms to predict alcohol dependence status with blood transcriptomic data. While these results are intriguing, the authors should submit their work to alcohol or addiction relevant journals instead of PLoS Computational Biology as all analyses in this study are conventional and do not involve any methods/algorithms/tools development.**

We thank the Reviewer for their comments and for finding the results intriguing. The journal’s scope is stated on its website: “*PLOS Computational Biology* features works of exceptional significance that further our understanding of living systems at all scales—from molecules and cells, to patient populations and ecosystems—through the application of computational methods.” We respectfully disagree that this manuscript is not suitable for PLoS Computational Biology as it is within the scope of the journal as stated on their website, and we believe it would be of interest to the journal’s readership.

**Major comments:
1. One of the major goals of this study was to compare the transcriptional response to alcohol dependence between the blood and different brain regions. However, the authors only provide Venn diagrams (Figure 2B) to show the overlap between differentially expressed genes (DEGs) in blood and brain regions without taking into consideration the magnitude of change in gene expression (e.g., fold change). This can be misleading because the analysis used a loose cutoff (see comment #2).**

We understand the Reviewer’s concern regarding the relatively small magnitude of fold changes observed and the permissive cutoff for DEGs (see the Reviewer’s second point). However, brain gene expression is very tightly regulated and relatively resistant to large changes in gene expression, so our results are expected and consistent with the fold changes and p-values observed in other brain transcriptome studies including those after CIE treatment. For example, Osterndorff-Kahanek and colleagues (14) analyzed the effects of CIE on brain gene expression in the amygdala, prefrontal cortex, and nucleus accumbens of male C57BL/6J mice. They found 604 DEGs in the PFC, 566 DEGs in the nucleus accumbens, and 400 DEGs in the amygdala between CIE and control mice (unadjusted p-value < 0.05) with fold changes between -1.17 and 1.80 in PFC, -1.13 and 1.51 in NAC, and -1.15 and 1.49 in amygdala. Notably, the fold changes of the DEGs identified in the present study range from -2.5 to 3.3. This is just one of many examples in the literature of brain gene expression studies.

It should be noted that even small changes in critical mRNAs are important, and the collective effects of small changes could be profound especially in heterogeneous tissues like brain and blood.

Most importantly, our main findings are based on gene networks and the probability of many genes in a network being a false positive is low. We do not believe this limitation greatly affects our conclusions because we do not emphasize individual genes. Rather, we based our conclusions on multiple levels of evidence.

**2. For the DE analysis, no multiple testing control and a very loose cutoff level (nominal p < 0.05) were used. The reviewer suspects that there is a very low overlap between DE genes in blood and brain regions if adjusted p.value < 0.01 is applied.**

The Reviewer is correct that there would be low overlap between the DEGs in blood and brain if adjusted p-value < 0.01 was applied, because only a few genes pass this stringent threshold. Similar to the small fold changes observed in brain gene expression datasets, the inability to pass stringent p-value thresholds is expected for brain gene expression data and consistent with previous studies including the one referred to above. In the study mentioned above, no genes in the PFC and amygdala were DE at adjusted p-value < 0.01 (and only 2 in the accumbens) (14). We have previously validated many genes which do not pass multiple testing correction on a different platform (RT qPCR) (18).

As noted in our response to the Reviewer’s first point, we based our conclusions on multiple lines of evidence and gene networks rather than individual genes. We therefore do not believe this limitation greatly affects our conclusions.

**3. It is not clear how the authors performed co-expression module preservation between blood and brain regions. Why not using the module preservation Z-summary statistics provided by WGCNA?**

We apologize if our methods were not clear in the manuscript and have now added text detailing how we determined whether coexpression modules were preserved between blood and brain.

“In addition to comparing the DEGs, we also compared the gene coexpression modules that were related to CIE between blood and brain following previously described approaches (19-23). Briefly, for each pair of networks, the overlap between all possible pairs of modules was calculated, and the significance of module overlap was assessed using a one-sided hypergeometric test. Blood and brain modules sharing a significant number of genes (hypergeometric p < 0.05), were considered conserved and we refer to them as blood-brain modules throughout the manuscript. The software Cytoscape (http://www.cytoscape.org/) was used to visualize the comparisons and create a meta-network of highly overlapping CIE modules.”

There are several ways to assess module preservation across conditions. One approach is using the Z-summary statistic from the WGCNA package as the Reviewer mentioned, which is a permutation-based method and is commonly used to determine whether the modules identified within a coexpression network are reproducible and reliable (24). Another method (and the one we present in the manuscript) is a cross-tabulation method performed as described above. Our group and others have used this approach to identify coexpression preservation (including Steve Horvath, one of the creators of the WGCNA package) (19-23). Another approach involves constructing a single network comprised of genes across *all* tissues (rather than constructing separate networks for each tissue and comparing the separate network modules), and then identifying modules that contain genes from multiple tissues (25). To be thorough, we used all three of these approaches and found that although there were some nuanced findings depending on the technique employed, all three analyses revealed similar conclusions. Given that similar conclusions were derived from all three analyses, we chose to only present the cross-tabulation analysis because it is highly intuitive and the most interpretable due to its simplicity. Reviewer #1 noted that “the results are presented in a thorough and clear fashion” which is much appreciated as we put great effort into presenting Results as clearly as possible without compromising the accuracy of the reporting.

**4. For the three machine-learning algorithms, it is not clear how many features were selected and what feature selection methods were used?**

We used all features (i.e., genes) to train the models, and the built-in feature selection of the random forest and logistic regression with regularization models reduced the genes in the final models. Some of the features had coefficients that went to zero so these genes fell out of the regularized logistic regression model. For random forest, some of the genes had importance scores (Mean Decrease in Gini) of zero and those genes did not contribute to the final model. We reported the number of features that were selected in the final model in the Results section. PLS-DA does not have built-in feature selection (although it does provide measures of feature importance which is partly why we chose the technique – please see the Variable Importance Measures section in the manuscript) and all genes were included in this model. We have clarified this in the Results:

“We used all genes to train the classifiers. Some of the coefficients in the regularized LR model go to zero and those features fall out of the model. Some features in the RF model have zero Mean Decrease in Gini metric and are not included in the final model. In this way the regularized LR and RF methods include embedded feature selection. The PLSDA model includes variable importance measures but does not include built-in feature selection and uses all genes for the model.”

**Have the authors made all data and (if applicable) computational code underlying the findings in their manuscript fully available?**
The [PLOS Data policy](https://journals.plos.org/ploscompbiol/s/materials-and-software-sharing) requires authors to make all data and code underlying the findings described in their manuscript fully available without restriction, with rare exception (please refer to the Data Availability Statement in the manuscript PDF file). The data and code should be provided as part of the manuscript or its supporting information, or deposited to a public repository. For example, in addition to summary statistics, the data points behind means, medians and variance measures should be available. If there are restrictions on publicly sharing data or code —e.g. participant privacy or use of data from a third party—those must be specified.

Reviewer #1: Yes

Reviewer #2: Yes

Reviewer #3: **No:** Not applicable

PLOS authors have the option to publish the peer review history of their article ([what does this mean?](https://journals.plos.org/ploscompbiol/s/editorial-and-peer-review-process#loc-peer-review-history)). If published, this will include your full peer review and any attached files.


If you choose “no”, your identity will remain anonymous but your review may still be made public.


**Do you want your identity to be public for this peer review?** For information about this choice, including consent withdrawal, please see our [Privacy Policy](https://www.plos.org/privacy-policy).

Reviewer #1: No

Reviewer #2: No

Reviewer #3: **Yes:** Xusheng Wang

1.	Tebani A, Gummesson A, Zhong W, Koistinen IS, Lakshmikanth T, Olsson LM, et al. Integration of molecular profiles in a longitudinal wellness profiling cohort. Nature communications. 2020;11(1):4487.

2.	Ferguson LB, Patil S, Moskowitz BA, Ponomarev I, Harris RA, Mayfield RD, et al. A Pathway-Based Genomic Approach to Identify Medications: Application to Alcohol Use Disorder. Brain Sci. 2019;9(12).

3.	Koob GF. Neurocircuitry of alcohol addiction: synthesis from animal models. Handb Clin Neurol. 2014;125:33-54.

4.	Koob GF, Volkow ND. Neurocircuitry of addiction. Neuropsychopharmacology : official publication of the American College of Neuropsychopharmacology. 2010;35(1):217-38.

5.	Daskalakis NP, Cohen H, Cai G, Buxbaum JD, Yehuda R. Expression profiling associates blood and brain glucocorticoid receptor signaling with trauma-related individual differences in both sexes. Proceedings of the National Academy of Sciences of the United States of America. 2014;111(37):13529-34.

6.	de Jong S, Boks MP, Fuller TF, Strengman E, Janson E, de Kovel CG, et al. A gene co-expression network in whole blood of schizophrenia patients is independent of antipsychotic-use and enriched for brain-expressed genes. PloS one. 2012;7(6):e39498.

7.	Glatt SJ, Everall IP, Kremen WS, Corbeil J, Sasik R, Khanlou N, et al. Comparative gene expression analysis of blood and brain provides concurrent validation of SELENBP1 up-regulation in schizophrenia. Proceedings of the National Academy of Sciences of the United States of America. 2005;102(43):15533-8.

8.	Hensman Moss DJ, Flower MD, Lo KK, Miller JR, van Ommen GB, t Hoen PA, et al. Huntington's disease blood and brain show a common gene expression pattern and share an immune signature with Alzheimer's disease. Scientific reports. 2017;7:44849.

9.	Mina E, van Roon-Mom W, Hettne K, van Zwet E, Goeman J, Neri C, et al. Common disease signatures from gene expression analysis in Huntington's disease human blood and brain. Orphanet J Rare Dis. 2016;11(1):97.

10.	van Heerden JH, Conesa A, Stein DJ, Montaner D, Russell V, Illing N. Parallel changes in gene expression in peripheral blood mononuclear cells and the brain after maternal separation in the mouse. BMC Res Notes. 2009;2:195.

11.	Paxinos G, Franklin KBJ. Paxinos and Franklin's the mouse brain in stereotaxic coordinates. 4th ed. ed. Amsterdam ;: Elsevier Academic Press; 2013.

12.	McKenzie AT, Wang M, Hauberg ME, Fullard JF, Kozlenkov A, Keenan A, et al. Brain Cell Type Specific Gene Expression and Co-expression Network Architectures. Scientific reports. 2018;8(1):8868.

13.	Lopez MF, Becker HC. Effect of pattern and number of chronic ethanol exposures on subsequent voluntary ethanol intake in C57BL/6J mice. Psychopharmacology. 2005;181(4):688-96.

14.	Osterndorff-Kahanek EA, Becker HC, Lopez MF, Farris SP, Tiwari GR, Nunez YO, et al. Chronic ethanol exposure produces time- and brain region-dependent changes in gene coexpression networks. PloS one. 2015;10(3):e0121522.

15.	Smith ML, Lopez MF, Archer KJ, Wolen AR, Becker HC, Miles MF. Time-Course Analysis of Brain Regional Expression Network Responses to Chronic Intermittent Ethanol and Withdrawal: Implications for Mechanisms Underlying Excessive Ethanol Consumption. PloS one. 2016;11(1):e0146257.

16.	Saarela M, Jauhiainen S. Comparison of feature importance measures as explanations for classification models. SN Applied Sciences. 2021;3(2):272.

17.	Litten RZ, Bradley AM, Moss HB. Alcohol biomarkers in applied settings: recent advances and future research opportunities. Alcoholism, clinical and experimental research. 2010;34(6):955-67.

18.	Maiya R, Pomrenze MB, Tran T, Tiwari GR, Beckham A, Paul MT, et al. Differential regulation of alcohol consumption and reward by the transcriptional cofactor LMO4. Mol Psychiatry. 2021;26(6):2175-86.

19.	Oldham MC, Konopka G, Iwamoto K, Langfelder P, Kato T, Horvath S, et al. Functional organization of the transcriptome in human brain. Nature neuroscience. 2008;11(11):1271-82.

20.	Miller JA, Horvath S, Geschwind DH. Divergence of human and mouse brain transcriptome highlights Alzheimer disease pathways. Proceedings of the National Academy of Sciences of the United States of America. 2010;107(28):12698-703.

21.	Ferguson LB, Zhang L, Kircher D, Wang S, Mayfield RD, Crabbe JC, et al. Dissecting Brain Networks Underlying Alcohol Binge Drinking Using a Systems Genomics Approach. Molecular neurobiology. 2018.

22.	Ferguson LB, Zhang L, Wang S, Bridges C, Harris RA, Ponomarev I. Peroxisome Proliferator Activated Receptor Agonists Modulate Transposable Element Expression in Brain and Liver. Front Mol Neurosci. 2018;11:331.

23.	Ponomarev I, Wang S, Zhang L, Harris RA, Mayfield RD. Gene coexpression networks in human brain identify epigenetic modifications in alcohol dependence. The Journal of neuroscience : the official journal of the Society for Neuroscience. 2012;32(5):1884-97.

24.	Langfelder P, Luo R, Oldham MC, Horvath S. Is my network module preserved and reproducible? PLoS computational biology. 2011;7(1):e1001057.

25.	Talukdar HA, Foroughi Asl H, Jain RK, Ermel R, Ruusalepp A, Franzén O, et al. Cross-Tissue Regulatory Gene Networks in Coronary Artery Disease. Cell systems. 2016;2(3):196-208.
